# Antibacterial and Cytotoxicity of Root Canal Sealer with the Addition of Chitosan Nanoparticle at Various Concentrations

**DOI:** 10.1055/s-0042-1746415

**Published:** 2022-06-21

**Authors:** Diatri Nari Ratih, Ema Mulyawati, Rika Kurnia Santi, Yulita Kristanti

**Affiliations:** 1 Department of Conservative Dentistry, Faculty of Dentistry, Universitas Gadjah Mada, Yogyakarta, Indonesia; 2 Former Student Specialist, Study Program of Conservative Dentistry, Faculty of Dentistry, Universitas Gadjah Mada, Yogyakarta, Indonesia Faculty of Dentistry, Universitas Gadjah Mada, Yogyakarta, Indonesia

**Keywords:** antibacterial, chitosan nanoparticle, cytotoxicity, root canal sealer

## Abstract

**Objectives** The aim of this study was to evaluate the addition of chitosan nanoparticle with concentrations of 0, 10, 20, and 30% to the epoxy resin-based (ERB) sealer on its antibacterial and cytotoxicity effect.

**Methods and Materials** This research was divided into two studies, the first study was the addition of chitosan with a concentration of 0% (as control), 10, 20, and 30% to an ERB sealer on its antibacterial effect, and the second study was on its cytotoxicity. An agar diffusion test was employed to determine the antibacterial effect on *Enterococcus faecalis* . An MTT (3-{4,5-dimethylthiazol-2-yl}-2,5-diphenyl tetrazolium bromide) assay was utilized to test the cytotoxicity by evaluating cell viability.

**Statistical Analysis** One-way analysis of variance and Tukey's test ( *α* = 0.05) were used to analyze data obtained from each evaluation with a significance level of 95%.

**Results** The addition of chitosan nanoparticles at concentrations 10, 20, and 30% produced a greater inhibition zone of *E. faecalis* ( *p* < 0.05), however, had less cytotoxicity compared with no addition of chitosan (0%) ( *p* < 0.05).

**Conclusion** The addition of chitosan nanoparticles at concentrations 10, 20, and 30% to the ERB sealer produced greater antibacterial and less cytotoxicity compared with no addition of chitosan (0%).

Root canal obturation is an important aspect that influences the successful of root canal treatment. Root canal sealer as material for obturation should have biocompatibility, antibacterial, good apical closure ability, adequate flow, insoluble in tissue fluid, and not staining teeth. 1 Root canal sealer must enable to eradicate the remains of microorganisms that cannot be removed during the root canal preparation and sterilization procedure. Of the numerous types of microorganisms, *Enterococcus faecalis* is a bacteria that is mostly found in the failure of endodontic cases. 2

In clinical conditions, the obturation of the root canal can be excessive, resulting in contacting of root canal sealers with the periapical tissue. The tissue response to the material can impair the result of root canal treatment. 3 Therefore, the biocompatibility of the sealer is essential for successful root canal treatment since the release of sealer substances can induce reactions in the periapical tissue. 4

Currently, the epoxy resin-based (ERB) sealer is popular as obturation material due to several beneficial properties such as good apical closure, good flow, low setting time, solubility, and inexpensive. 5 However, previous studies reported that resin sealers have low antibacterial properties; hence, the addition of antibacterial components to sealers has the potential to increase their antibacterial efficacy. 6 7

Lately, chitosan has been frequently used in the health field for its many advantageous properties. Chitosan is a nontoxic natural polysaccharide, produced from the deacetylation of chitin obtained from the shells of crustaceans. Chitosan broadly exists in nature, is inexpensive, and possesses chelating properties. 8 Former studies have explained that chitosan yielded antibacterial properties against *E. faecalis* and *Candida albicans* . 9 10 Due to the many advantages of chitosan, hence chitosan nanoparticles are incorporated into ERB sealer to enhance its antibacterial efficacy. Previous studies have shown that the addition of chitosan nanoparticles to ERB sealer did not affect the physical properties of ERB sealer, which exhibited acceptable sealer for obturation material. 11 12 13

However, until now there is deliberation about the appropriate concentration of chitosan nanoparticles, which should be added to sealer. Thus, the purpose of this study was to evaluate the addition of nanoparticle chitosan with concentrations of 0, 10, 20, and 30% to the ERB sealer on its antibacterial and cytotoxicity effect. The null hypothesis was that no difference occurred in antibacterial and cytotoxicity effect with the addition of nanoparticle chitosan with concentrations of 0, 10, 20, and 30% to the ERB sealer.

## Materials and Methods

The research protocol was approved by the institutional ethics committee under the number 00483/KKEP/FKG-UGM/EC/2020. This study evaluated the addition of chitosan nanoparticles (NHI, Tangerang, Indonesia) with concentrations of 0 (as control), 10, 20, and 30% to ERB sealer (AH 26, De Trey, Dentsply, Konstanz, Germany), and was divided into two evaluations, namely, antibacterial and cytotoxicity test.

### Antibacterial Evaluation

The antibacterial study used 24 samples assigned into 4 groups of each 6 samples. The methodology for evaluating the antibacterial effect was performed using agar diffusion test (ADT). The procedure for the ADT was modified from Silva et al. 14 A 24-hour culture of *E. faecalis* (strain ATCC 29212) in Brain Heart Infusion agar was employed to make a bacterial suspension encompassing 108 viable bacterial cells per milliliter. Standardization of 0.5 suspensions with a spectrophotometer and McFarland scale was then performed. *E. faecalis* suspension was planted using a sterile swab on Muller Hinton Agar plates and incubated for 24 hours at 37°C. Afterward desiccating for 10 minutes at 36°C, a sterile glass tube was employed to produce 4 agar wells (6 mm diameter × 4 mm depth) of each petri.

**Fig. 1:**
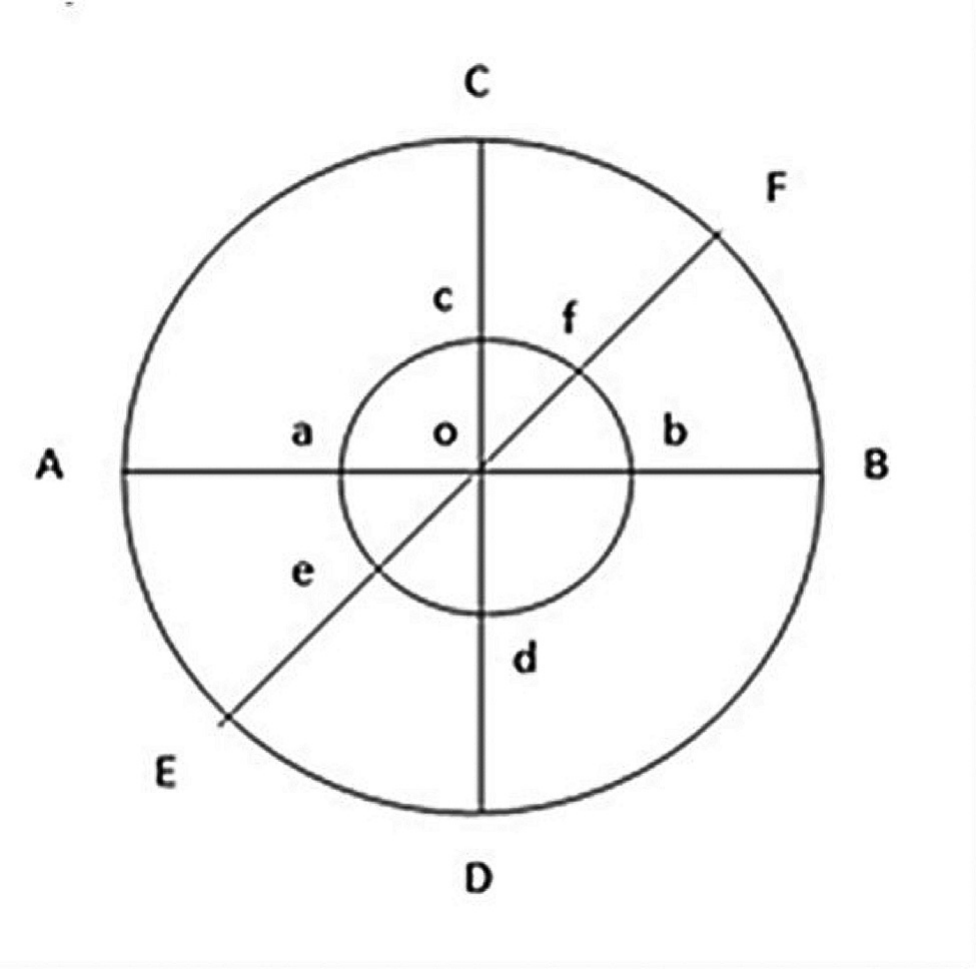
The measurement of inhibition zone; Point O: center point of the well; Line AB, CD, and EF: diameter of radical zone; Line ab, cd, and ef: diameter of the well (6 mm).

The wells were filled with the materials described as follows: group 1: 0% chitosan nanoparticle + ERB sealer as a control group, group 2: 10% chitosan nanoparticles + ERB sealer, group 3: 20% chitosan nanoparticles + ERB sealer, and group 4: 30% chitosan nanoparticles + ERB sealer. Before placing the material into the well of each petri, the sealer was mixed concordant to the instruction of the manufacturer until a homogeneous consistency was obtained. All sealers were then put in the well according to their respective groups. Furthermore, all petri were stored in an incubator at 37°C for 24 hours. After incubation, the zones of microbial growth inhibition were assessed at the radical zone using a sliding caliper on a millimeter scale with a precision of 0.02 mm, based on Fig. 1 and the formula developed by Levinson. 15

The measurement of radical zone of the well: ½ (AB – ab) + ½ (CD – cd) + ½ (EF – ef)/3.

### Cytotoxicity Evaluation

#### Samples Preparation

All samples were created according to the International Organization for Standardization (ISO) 10993–12. 16 This evaluation used 48 samples divided into four concentration groups of chitosan nanoparticles as in antibacterial evaluation. Each concentration group consisted of 12 samples. The mixed sealers were located into 48 Teflon molds (5 mm in diameter and 2 mm in height), permitted to set in an incubator at 37°C for 24 hours, crushed into tiny particles with mortar and pestle, and located in the cell culture dish.

#### Cell Culture

This present study used Vero cells (ECACC, Public Health England, London, U.K.) for cytotoxicity evaluation, which was attained from Cell Culture Laboratory, LPPT UGM, Yogyakarta, Indonesia. The procedure for the cytotoxicity evaluation was adapted from Catunda et al. 17 The cells were cultured in M199 medium (Gibco, Waltham, MA) at 37°C in a humidified 5% CO _2_ atmosphere. The culture medium utilized was Dulbecco's modified Eagle medium (DMEM, Sigma Chemical Co., St Louis, MO) accompanied with 10% fetal bovine serum (Sigma Aldrich, St Louis, MO) and 1% antibiotic-antimycotic solution (10,000) UI of penicillin, 10 mg of streptomycin in 0.9% sodium chloride (Sigma Chemical Co.). Cultures were delivered with fresh medium every 3 days until an adequate number of cells was attained. Following dilution with a ratio of 1:10 in Trypan Blue Dye (10 µL of cells in 90 µL of Trypan Blue), the cells were calculated in a Neubauer chamber. The cells (2 × 105 cells/mL of DMEM per well) were then moved to the culture plate (Sigma-Aldrich, Munich, Germany) and incubated for 24 hours at 37°C in 5% CO _2_ and 95% air. All samples were prepared by the same operator in a laminar flow chamber (Biobase, Jinan, Shandong, China), and exposed to ultraviolet light for 45 minutes.

#### Extracts and Experimental Groups

The extract was made by submerging the sample in DMEM kept in a Falcon tube for 24, 48, or 72 hours to condition the media. The conditioned DMEM was filtered (0.22 m syringe filter; TPP, Darmstadt, Germany) to eradicate dense components.

#### Cytotoxicity Assay

In 96-well culture plates (Thermo-Fisher Scientific, Waltham, MA), 2 × 105 cells in 1 mL of DMEM per well were cultured and grown to subconfluent monolayers for 24 hours. The culture medium was then altered with equal volumes (25 µL) of sealer extracts (conditioning medium), using the culture medium itself as a negative control. The assessment of the cytotoxic activity was substantiated by the colorimetric method bromide (3-{4,5-dimethylthiazol-2-yl}-2,5-diphenyl tetrazolium bromide) (MTT). After 24 hours of incubation, 25 µL (5 mg/mL) of MTT solution was inserted to each well, and the plates were incubated for 3 hours. The MTT was then detached and 25 µL per well dimethyl sulfoxide (Sigma Chemical Co.) was put into each well to dissolve the formazan crystals.

Based on ISO 10993–12, 16 a reduction in the number of alive cells leads to a decline in the metabolism in the sample. Such reduction is directly associated with the quantity of blue-violet formazan created as observed by the optical density at 570 nm using enzyme-linked immunosorbent assay reader (Tecan Spark, Tecan Trading AG, Switzerland). The percentage of viable cells in each well was calculated as below 18 :

#### Absorbance of Sample

% Cell viability = (Absorbance of sample/Absorbance of control) × 100%.

#### Absorbance of Control

The lesser viability % value means higher cytotoxic potential. The cell viability was categorized as noncytotoxic (more than 90% cell viability), slightly cytotoxic (60–90% cell viability), moderately cytotoxic (30–59% cell viability), and severely cytotoxic (less than 30% cell viability). 19

### Statistical Analysis

Data obtained from each evaluation were assessed separately using the Shapiro–Wilk normality test for normal data distribution and Levene's test for homogeneity of variances. Then, each evaluation data were analyzed using analysis of variance (ANOVA), followed by Tukey's test with *p* < 0.05 considered as a significant difference. The statistical analysis was processed and analyzed using the SPSS Version 23 program.

## Results

**Fig. 2:**
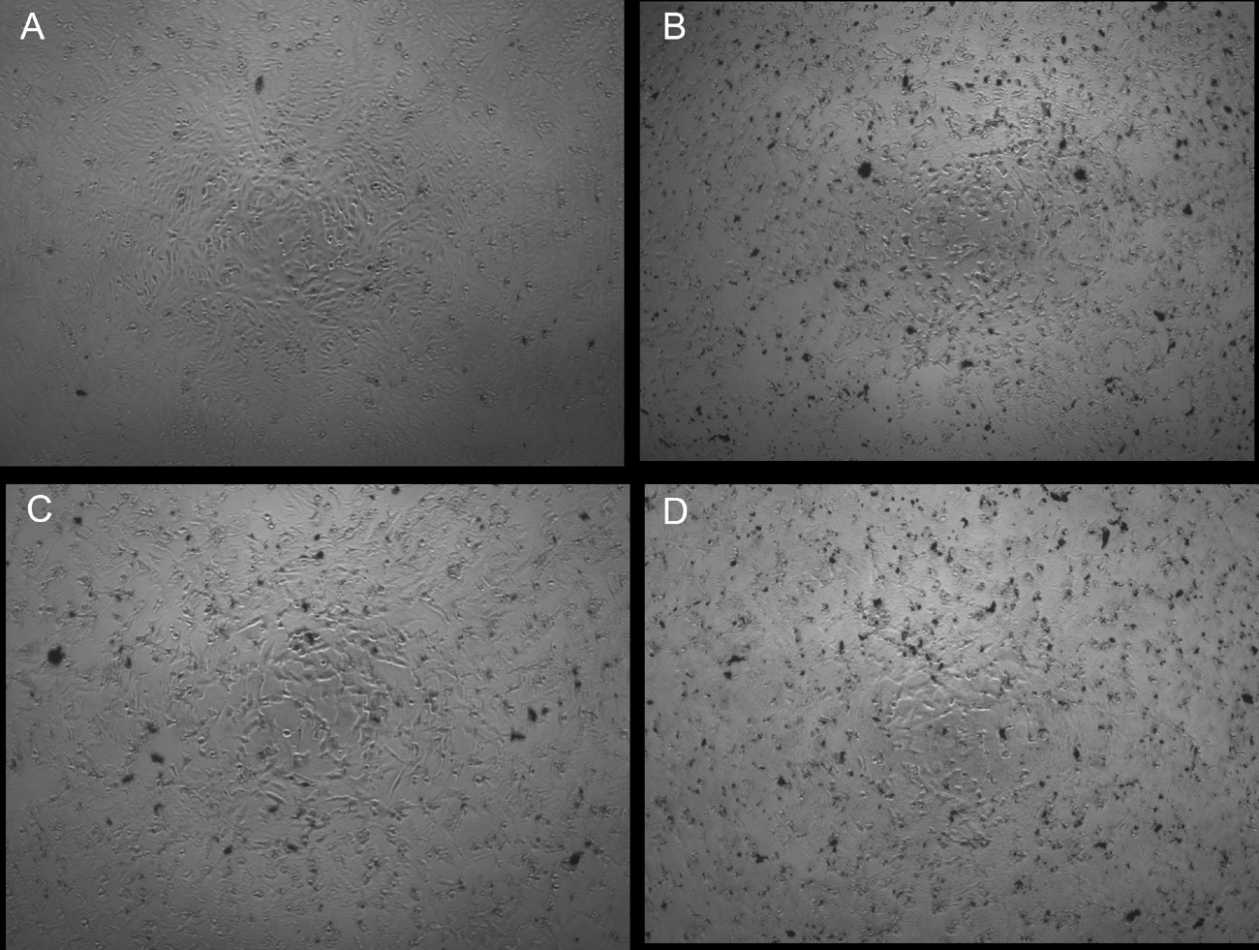
Micrographs showed cell viability of chitosan nanoparticles addition at concentration 0% ( **A** ), 10% ( **B** ), 20% ( **C** ), and 30% ( **D** ) to epoxy resin-based sealer. The quantity of formazan (related to the number of viable cells) is showed as black spot as monitored by the optical density (OD) at 570 nm.

The mean inhibition zone diameter of *E. faecalis* was the highest at 10% concentration and the lowest was at 0% concentration, whereas the greatest toxicity was at 0% concentration and the least toxicity was at 30% concentration ( Table 1 ). The cell viability of representative samples can be seen in Fig. 2 , which exhibits the more the quantity of formazan that occurred (apparently directly proportional to the number of viable cells) the more the cell viability.

**Table 1:** Mean and standard deviation of *Enterococcus faecali* *s* inhibition zone and cell viability of chitosan nanoparticles addition at concentration 0, 10, 20, and 30% to epoxy resin-based sealer

Chitosan nanoparticles concentrations	*Enterococcus faecalis* inhibition zone (mm)	Cell viability (%)
0%	4.82 ± 0.54 ^a^	61.90 ± 1.20 ^a^
10%	9.94 ± 0.63 ^b^	73.08 ± 1.07 ^b^
20%	9.63 ± 0.35 ^b^	74.08 ± 0.90 ^c^
30%	9.60 ± 0.25 ^b^	75.42 ± 1.43 ^c^

Note: Different letters indicate that there were statistically significant differences.

One-way ANOVA analysis exhibited that the addition of chitosan nanoparticles at concentrations 10, 20, and 30% to ERB sealer generated a greater inhibition zone of *E. faecalis* but less cytotoxicity than no addition of chitosan (0%) ( *p* < 0.05). Tukey's test revealed that the addition of chitosan nanoparticles at concentrations 10, 20, and 30% caused significant increase in antibacterial efficacy to *E. faecalis* compared with no addition of chitosan nanoparticles ( *p* < 0.05); however, no significant differences occurred among concentrations 10, 20, and 30% ( *p* > 0.05). The addition of chitosan nanoparticles at concentrations 10, 20, and 30% to the EBS sealer produced less cytotoxicity compared with concentration 0% ( *p* < 0.05), while between 20 and 30% concentrations, no significant difference occurred ( *p* > 0.05).

## Discussion

The most crucial requirements of sealers for root canal obturation are antibacterial and biocompatible; therefore, this study was conducted to evaluate the antibacterial effect and biocompatibility with cytotoxicity test. 20 Previous studies stated that the ERB sealer has a minimal antibacterial property. 5 However, in the clinical condition, this minimal antibacterial property of ERB sealer is inadequate to protect against persistent bacterial infections such as *E. faecalis* since this microorganism is usually related to the etiology of tenacious periradicular lesions. 21 Additionally, in most studies of chitosan, the antibacterial efficacy of chitosan nanoparticles has already been proven 6 9 14 ; therefore, in this study, it was verified that chitosan nanoparticles with the concentration of 10, 20, and 30% added to ERB sealer would enhance the antibacterial efficacy of ERB sealer.

The efficacy of chitosan to eliminate microorganisms can be explained by two mechanisms. The first mechanism is that chitosan nanoparticles bind to the target cell membrane through electrostatic forces resulting in changes in the membrane, depolarization, and loss of membrane integrity. The important cell functions of the bacteria are disrupted such as respiration, nutrient transport, and energy transduction resulting in bacterial cell death. The second mechanism is the production of free radicals such as reactive oxygen species which can affect the resistance of bacterial cells by inhibiting protein function and damaging deoxyribonucleic acid. 22

This present study revealed the development of the inhibition zone of ERB sealer also occurred with no addition of chitosan nanoparticles. It might be due to the presence of antibacterial components, such as hexamethylenetetramine and formaldehyde in the ERB sealer used in this study (AH 26). 23 The more concentration addition of the chitosan to ERB sealer may not affect the antibacterial activity as shown in this study. 24

ADT was selected to evaluate antibacterial properties in this study since this technique has been widely used to evaluate the antibacterial activity of sealers and offers many advantages, such as simplicity, low cost, the ability to test enormous numbers of microorganisms and antimicrobial agents, and the ease to interpret results provided. 25 In addition, this method allows measurement of the activity of soluble and degradable components of the tested material, such as chitosan nanoparticles used in this study incorporated to ERB in the surrounding medium, indicated by an inhibition halo. 26

Besides antibacterial properties, sealers should be biocompatible since they enable to contact with periapical tissue. 4 This study showed that all concentrations of chitosan addition produced a cytotoxic effect, although with different degrees of toxicity, and all concentrations including without the addition of chitosan were classified as slightly cytotoxic (cell viability ranged from 60 to 90%). 19 ERB sealer without addition of chitosan (0%) produced the highest cytotoxicity compared with the addition of other concentrations of chitosan nanoparticles. It can be explained that the ERB sealer employed in this study (AH 26) consisted of formaldehyde, epoxy resin, and hexamethylenetetramine, besides being antibacterial, they are also toxic. 23 27

This cytotoxic effect in this study was only observed after 24 hours. This effect may decline if the longer observation is undertaken because the release of formaldehyde is reduced on the 7th day. 28 The cytotoxic influence may also be associated with the solubility of the material used, thus the leaching out of sealer components is influenced by its solubility. 29 In this study, the addition of 30% chitosan nanoparticles induces the lowest cytotoxic effect, perhaps the more chitosan, which is biocompatible in nature, added to the ERB sealer generated less quantity of the cytotoxicity components (formaldehyde, epoxy resin, and hexamethylenetetramine) in the sealer mixture. Consequently, the leaching of the cytotoxic components also decreased. 30

## Conclusion

The addition of chitosan nanoparticles at concentrations 10, 20, and 30% to the ERB sealer produced greater antibacterial and less cytotoxicity compared with no addition of chitosan (0% concentration).
